# Diagnostic performance of contrast-enhanced multidetector computed tomography and gadoxetic acid disodium-enhanced magnetic resonance imaging in detecting hepatocellular carcinoma: direct comparison and a meta-analysis

**DOI:** 10.1007/s00261-016-0807-7

**Published:** 2016-06-18

**Authors:** Jin Guo, Youngkwon Seo, Shuo Ren, Sunwoo Hong, Dongki Lee, Soyoun Kim, Yuanyuan Jiang

**Affiliations:** 1 Department of Medical Biotechnology, Dongguk University, Seoul, Korea; 2 Department of Anesthesia, Sun Yat-sen University Cancer Center, Guangzhou, Guangdong People’s Republic of China; 3 Department of Chemistry, Sungkyunkwan University, Suwon, Korea

**Keywords:** Hepatocellular carcinoma, Diagnosis, Computed tomography, Gadoxetic acid disodium-enhanced magnetic resonance imaging, Meta-analysis

## Abstract

The purpose of this study was to directly (head-to-head) compare the per-lesion diagnostic performance of contrast-enhanced computed tomography (CT) (also referred to as CT hereafter) and gadoxetic acid disodium (Gd-EOB-DTPA)-enhanced magnetic resonance (MR) imaging (also referred to as MRI hereafter) for the detection of hepatocellular carcinoma (HCC). Studies reporting direct per-lesion comparison data of contrast-enhanced multidetector CT and Gd-EOB-DTPA-enhanced MR imaging that were published between January 2000 and January 2015 were analyzed. The data of each study were extracted. Systematic review, paired meta-analysis, and subgroup analysis were performed. Twelve studies including 627 patients and 793 HCC lesions were analyzed. The sensitivity estimates of MRI and CT were, respectively, 0.86 (95% CI 0.76–0.93) and 0.70 (95% CI 0.58–0.80), with significant difference (*P* < 0.05). The sensitivity estimates were both 0.94 (95% CI 0.92–0.96) (Chi-square 4.84, degrees of freedom = 1, *P* > 0.05). In all subgroups, Gd-EOB-DTPA-enhanced MR imaging was more sensitive than multidetector CT for the detection of HCC, and specificity estimates of both tests maintained at a similarly high level in all conditions: sensitivity estimates of both tests were reduced in studies where patients were diagnosed with HCC solely by liver explant or in those where HCC lesions were small (≤2 cm, especially when ≤1 cm). But in all situations, sensitivities of MRI were higher than those of CT with or without significance. Gd-EOB-DTPA-enhanced MR imaging showed better per-lesion diagnostic performance than multidetector CT for the diagnosis of HCC in patients with cirrhosis and in small hepatic lesions.

**Electronic supplementary material:**

The online version of this article (doi:10.1007/s00261-016-0807-7) contains supplementary material, which is available to authorized users.

Hepatocellular carcinoma (HCC) is the second most common cause of cancer-related death worldwide. The estimated 782,000 new cases of HCC were diagnosed in 2012, of which 83% occurred in less developed areas [1]. Only patients with early-stage HCCs can undergo curative treatment, including liver resection, liver transplantation, and percutaneous local ablative treatment, according to the Barcelona Clinic Liver Cancer staging system [2]. Therefore, the accurate diagnosis of HCC is important to provide better treatment options for patients, especially in early-stage HCCs.

Effective non-invasive imaging techniques, including computed tomography (CT) and magnetic resonance (MR) imaging, can be used to diagnose HCC. According to the guidelines of the American Association for the Study of Liver Diseases (AASLD), a single dynamic technique showing intense arterial uptake followed by a ‘‘washout’’ of contrast in the venous-delayed phases is valid to diagnose HCC [3]. These guidelines have been adopted by the European Association for the Study of the Liver (EASL) and the European Organization for Research and Treatment of Cancer (EORTC) [4].

Because of its short acquisition time and high spatial resolution, CT is commonly used imaging technique for diagnosing HCC. However, MR imaging, which offers several beneficial characteristics such as the combination of various sequences and absence of X-ray radiation, is also often used independently or in combination with CT to improve the detection and diagnosis of HCC.

Despite research efforts aimed at identifying the optimal imaging technique, studies have shown a similar or slightly better diagnostic performance of dynamic MR imaging compared with multiphasic CT [5].

In recent years, the introduction of a new MR imaging contrast agent, gadolinium ethoxybenzyl diethylenetriamine pentaacetic acid (gadoxetic acid disodium or Gd-EOB-DTPA), may provide a solution. As a liver-specific contrast agent, it provides routine multiphasic information as well as tissue-specific physiological information during the hepatobiliary phase (HBP), thus improving the detection of HCC [6]. Several studies have compared the efficacy of Gd-EOB-DTPA-enhanced MR imaging (also referred to as MRI hereafter) to that of CT for the detection of HCC. Some studies suggested that Gd-EOB-DTPA-enhanced MR imaging shows better diagnostic performance than CT for HCC [7–11], whereas other studies suggested that there are no significant differences between the two techniques [12–16]. There is currently no consensus on the optimal method for the diagnosis of HCC, and to date, no meta-analysis has been conducted to clarify this issue.

In the present study, we performed a meta-analysis that included 12 studies selected according to strict criteria to estimate and compare the accuracy of Gd-EOB-DTPA-enhanced MR imaging to that of multidetector CT (also referred to as CT hereafter) for the diagnosis of HCC.

## Materials and methods

This meta-analysis was performed in accordance with the recommendations outlined in the Preferred Reporting Items for Systematic Reviews and Meta-Analyses statement (PRISMA) [17]. There is no review protocol registered for this research.

### Literature search

The MEDLINE, EMBASE, and Web of Science databases, and the Cochrane Library were searched for studies analyzing the per-lesion diagnostic accuracy of multidetector CT and Gd-EOB-DTPA-enhanced MR imaging for HCC in patients older than 18 years. The reference lists of the included original articles were manually checked to identify additional potential studies. Review articles and websites of major conferences were also searched. Table 1 shows the detailed search strategy and query terms.

**Table 1:** Literature search strategy

Step no.	Query
#1	(Liver OR hepatocellular) OR hepatic
#2	(Carcinoma OR tumor) OR cancer
#3	MeSH^a^ descriptor Carcinoma, Hepatocellular explode all trees
#4	“Computed tomography” OR “CT”
#5	MeSH descriptor Tomography, X-Ray Computed explode all trees
#6	“Magnetic resonance” OR “MR”
#7	MeSH descriptor Magnetic Resonance Imaging explode all trees
#8	(((“Gd-EOB-DTPA” OR “Gadolinium-EOB-DTPA”) OR “gadoxetic acid disodium”) OR eovist) OR primovist
#9	MeSH descriptor Gadoxetic Acid Disodium explode all trees
#10	(#1 AND #2) OR #3
#11	(#4 OR #5) AND (#6 OR #7)
#12	#8 OR #9
#13	#10 AND #11 AND #12 from January 2000 to January 2015

This table provides details on how the literature was searched in various databases

^a^Medical subject headings

### Inclusion and exclusion criteria

Inclusion and exclusion criteria were based on the participants, interventions, comparisons, outcomes, and study design. Studies were included if all of the following criteria were met: (a) the study was performed for per-lesion comparison; (b) patients were suspected of HCC based on prior ultrasound examination or alpha-fetoprotein, or diagnosed with HCC retrospectively; (c) sample size larger than ten patients; (d) diagnostic results of true positive (TP), false positive (FP), false negative (FN), and true negative (TN) were available; (e) both CT and MR imaging were performed by multiphasic contrast-enhanced imaging with Gd-EOB-DTPA as one of the contrast agents for MR imaging; (f) a CT detector row of at least four and an MR imaging magnetic field strength > 1.0T; and (g) reference standards were based on histopathology (explanted liver, liver resection, biopsy) and/or a clinical follow-up of at least 6 months. Studies were excluded if (a) any one of the inclusion criteria was not met; (b) multiple reports were published for the same study population, in which case only the most detailed and/or most recent publication was included. Discrepancies were resolved through discussion; and (c) articles published only as conference abstracts that did not include complete data or non-English language papers were not considered.

### Literature screening

Two reviewers independently screened the titles and abstracts and assessed the full text to identify potentially eligible papers. Papers were selected for review if they included patients with HCC who underwent both multidetector CT and Gd-EOB-DTPA-enhanced MR imaging for lesion evaluation.

### Quality assessment and data extraction

The quality assessment of diagnostic accuracy studies-2 (QUADAS-2) tool was used to evaluate the quality of all studies. The quality of primary diagnostic studies was assessed through estimation of risk of bias for four domains and clinical applicability for three domains of the study characteristics [18].

Meanwhile, a standardized excel form was used to extract relevant data from all studies, including author, year of publication, journal name, country of origin, type of study design (retrospective or prospective), clinical characteristics (age, gender, etiology of underlying chronic liver disease, proportions of cirrhosis, and Child-Pugh class), sample size (number of patients and number of HCC lesions), HCC lesion characteristics [lesion size, degree of tumor differentiation (well/moderately/poor), vascularity], reference standards, CT and MR imaging scanner type, number of CT rows, magnetic field strength of MR imaging, timing of arterial/venous/delayed phase imaging, descriptions of the interpretations of the diagnostic tests, interval between imaging readings of both index texts, imaging interpretation method (blinded or not, use of a confidence rating scale), and interval between pathology and imaging scanning. These data are presented in Table 2.

**Table 2:** Characteristics of the included studies

Study	Study design	Patient enrollment	Number of patients	Number of lesions	Gender ratio (m:f)	Patient age: mean (range)	Cirrhosis	Child-pugh class A/B/C	Number of HCC lesions	Lesion size (cm)
Kakihara et al.	Retrospective	ND^a^	15	118	11:4	55 (45–71)	Cirrhosis	0/8/7	61	1.3 (0.5–6.6)
Nishie et al.	Retrospective	ND	12	103	9:3	60 (46–72)	Cirrhosis	0/6/6	26	1.2 (0.5–3.4)
Yoo et al.	Retrospective	Consecutive	33	104	27:6	53.3 (46–60)	Cirrhosis	46/16/5	82	2.25 (0.3–5.1)
Di Martino et al.	Prospective	Consecutive	58	109	39:19	63 (35–84)	Cirrhosis	30/21/7	87	1.8 (0.3–7.0)
Akai et al.	Prospective	Consecutive	34	CT: 74 MR: 97	27:7	65 (48–78)	Cirrhosis or not	ND	52	2.6 (0.4–15.2)
Haradome et al.	Retrospective	Consecutive	75	99	60:15	54.7 (42–67)	Cirrhosis or not	48/5/1	60	1.74 (1.09–2.39)
Baek et al.	Retrospective	Consecutive	51	111	43:5	(32–80)	Cirrhosis	42/7/2	73	2.98 (0.2–10)
Sun et al.	Retrospective	ND	42	CT: 61 MR: 60	56:13	56 (39–73)	Cirrhosis	ND	33	1.37 (0.96–1.78)
Kim et al.	Retrospective	Consecutive	62	132	54:8	55 (31–67)	Cirrhosis or not	62/0/0	83	2.9 (0.5–10.5)
Sano et al.	Retrospective	Consecutive	64	CT: 248 MR: 253	47:17	m^b^: 66 (56–75); f^c^: 71 (62–80)	Cirrhosis or not	54/10/0	96	1.27 (0.4–2)
Granito et al.	Prospective	Consecutive	33	48	25:8	70 (48–74)	Cirrhosis	28/5/0	38	1.8 (1.0–3.0)
Park et al.	Retrospective	Consecutive	148	125	103:45	56 (30–73)	Cirrhosis	141/5/2	102	1.3 (0.6–2.0)

Study	Blind^d^	CT or MRI read first	Time interval between MRI and CT reading	CT Scanner row	Magnetic field strength	Use pathology as the only reference standard	Gd-EOB-DTPA-enhanced MR imaging				Multidetector CT			
							Tp^h^	Fp^i^	Fn^j^	Tn^k^	Tp	Fp	fn	tn
Kakihara et al.	Y	CT	4 weeks	64	1.5 T	Yes	20	2	16	80	15	3	21	79
Nishie et al.	Y	CT	4 weeks	64	3 T	Yes	16	4	14	69	16	4	14	69
Yoo et al.	Y	CT	ND	64	3 T	Yes	54	2	28	20	36	1	46	21
Di Martino et al.	Y^e^	Random	ND^g^	64	1.5 T	No	74	1	13	21	60	5	27	17
Akai et al.	Y^f^	Reader 1 CT, Reader 2 MR	>2 weeks	64	1.5 T	Yes	45	4	7	41	41	3	12	18
Haradome et al.	Y^e^	CT	3 weeks	16	1.5 T	No	52	3	8	36	42	2	18	37
Baek et al.	Y	Random	ND	64, 16, 4	3 T	No	65	2	8	34	56	2	17	36
Sun et al.	Y	MR	2 weeks	64, 16, 8	3 T	No	30	2	3	25	18	1	15	27
Kim et al.	Y	Random	4 weeks	64, 40, 16	3 T	Yes	77	2	6	47	74	3	9	46
Sano et al.	Y	Random	>1 weeks	16	1.5 T	Yes	87	8	4	154	58	3	29	158
Granito et al.	Y	ND	ND	6, 16	1.5 T	No	38	3	0	7	22	0	16	10
Park et al.	Y	CT	ND	64, 40	3 T	No	100	3	2	20	64	1	38	22

This table provides details of all the studies included in this research, from clinical information to imaging information

^a^No data

^b^Male

^c^Female

^d^Image readers were blinded to patient’s histories, laboratory results, findings of other imaging techniques, and final diagnosis

^e^Image readers were aware of patients’ history of cirrhosis and that patients had been suspected of HCC

^f^Image readers were aware that patients were suspected of HCC

^g^Reading session interval (where MR and CT images were mixed in a random order in one session) > 4 weeks

^h^True positive. The number in this column refers to the number of true-positive lesions

^i^False positive. The number in this column refers to the number of false-positive lesions

^j^False negative. The number in this column refers to the number of false-negative lesions

^k^True negative. The number in this column refers to the number of true-negative lesions

Values from each study were also extracted including categories of true positive, false positive, false negative, and true negative (Table 2). These values were used to generate a two-by-two contingency table showing the cross classification of disease status (result of the reference standard) and test outcome (result of the index test). Other statistical indexes, including positive likelihood ratio (PLR) and negative likelihood ratio (NLR), were also recorded if available. If there were more than two imaging evaluators, accuracy data were averaged among them (most of the researches provided the reading results of more than one image reader. In our work, in order to count the result in each study, these results of each reader in one study were average to stand for the results of one study). For studies including multiple technical aspects of the same imaging modality, data on the most advanced technique were extracted from the contingency table (e.g., data on MR imaging scanning with HBP combined with routine multiple phases were extracted instead of data on routine multiple phases alone).

### Statistical analysis

Forest plots of sensitivity and specificity were constructed using Review Manager (RevMan) (Version 5.3. Copenhagen: The Nordic Cochrane Centre, The Cochrane Collaboration, 2014). Parameters of bivariate model were externally calculated (see following) and were input to define the SROC curve. These plots were used to visually explore between-study variation in the diagnostic accuracy of each test.

We used the xtmelogit command in Stata version 13.0 for Mac (64-bit Intel) (Stata, College Station, TX) to fit the bivariate model to derive summary estimates of sensitivity, specificity, PLR, NLR, and their 95% Confidence Intervals (CIs). The paired sensitivity/specificity data for the tests were at level one of the analysis, and a binary covariate was created to identify each test in the two-by-two contingency table which had been generated from data extraction of each study. This model assumed that the sensitivities/specifies from individual studies (after logit transformation) are approximately normally distributed around a mean value with a certain amount of variability around this mean. It first transformed tp/tn/fp/fn into logit form with corresponding variance, and then using this logit form and its variance, the sensitivity, specificity, and their CIs can be calculated by Wald Chi-square test. Tests were compared by adding a covariate for test type to the bivariate model. Likelihood ratio tests were used to obtain the statistical differences between the sensitivities and specificities of the two tests by fitting alternative models (adding or removing covariate terms from the model). Subgroup analysis was performed in the following the same method [19].

Subgroup analysis was pre-specified to consider potential factors that could contribute to heterogeneity. Those factors included type of study design (prospective or retrospective), cirrhosis in patients (“cirrhosis”: all patients with cirrhosis; “cirrhosis or not”: part of patients with cirrhosis), mean size of HCC lesions, and reference standards (findings in explanted liver as the only reference or not). Meta-regression assists the decision making in subgroup analysis by revealing the most significant factors for heterogeneity. Therefore, subgroup analysis was then performed based on the identified factors. The first level of subgroup analysis was performed by comparing two groups divided by the fact that whether findings in liver explantation were used as the sole reference standard. Three of the studies used findings in the liver explantation as the sole reference standard [11, 14, 16], and the other nine studies had a composite standard of reference. The second level of subgroup analysis was performed in the subgroup of nine studies using a composite reference standard, by comparing five studies [7, 10, 13, 20, 21] which enrolled only patients with liver cirrhosis and those in which not all patients had liver cirrhosis.

Because lesion size is a substantial factor for the imaging diagnosis of HCC, subgroup analysis was independently performed according to lesion size, regardless of the meta-regression result. In this analysis, only sensitivity estimates were calculated because of the lack of true-negative values. We used 1 cm and 2 cm as cutoff values and compared the diagnostic sensitivities of the two imaging techniques. In this way, we had four subgroups of patients data separated extracted from studies: group 1 where lesions were equal or smaller than 1 cm, group 2 where lesions were larger than 1 cm, group 3 where lesions were equal or smaller than 2 cm, and group 2 where lesions were larger than 2 cm.

## Results

### Selection of studies

**Fig. 1:**
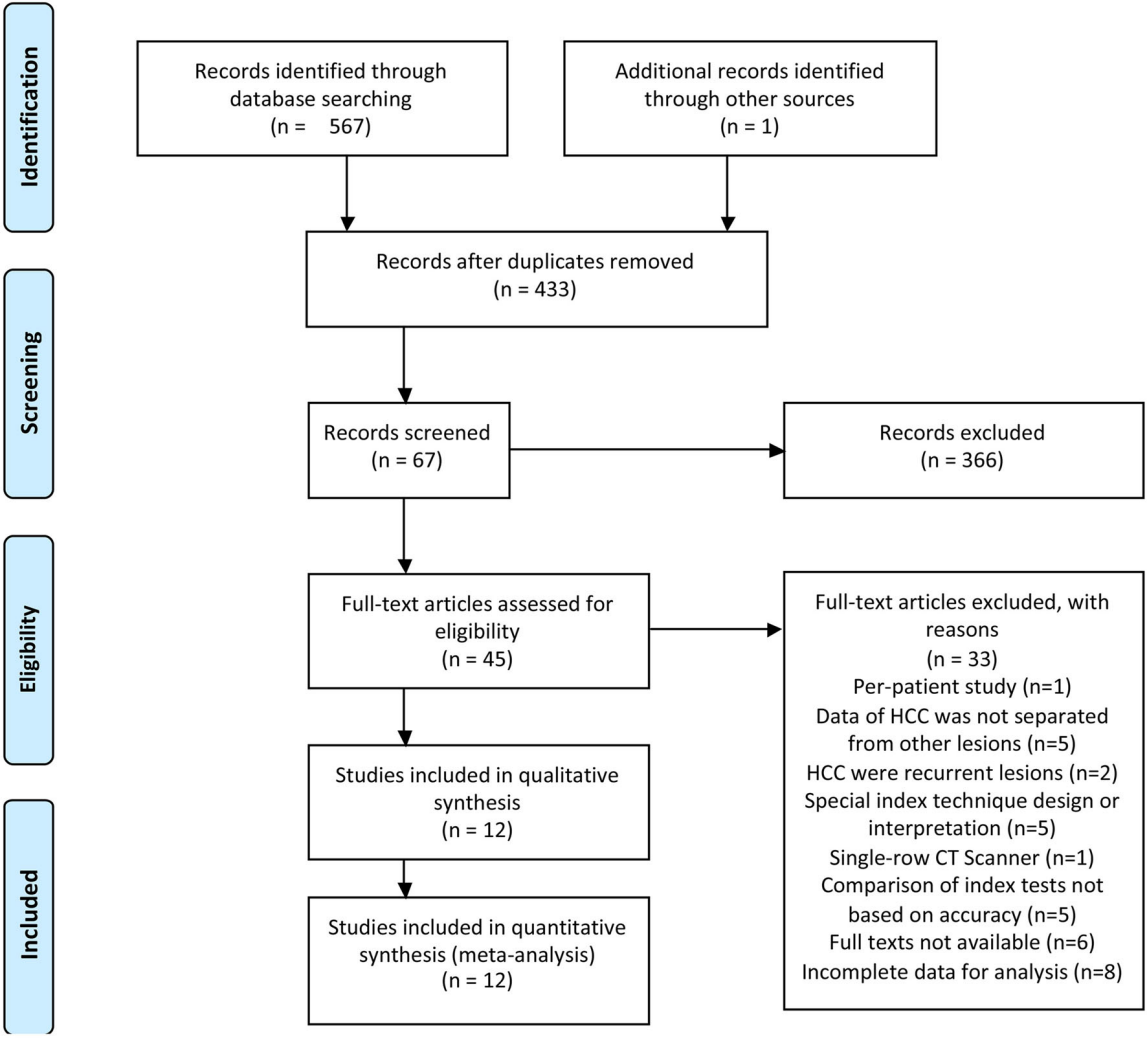
Flowchart illustrating the selection of studies

Multiple database searches initially yielded 568 potential literature citations, of which 67 were potentially relevant according to their titles and/or abstracts. Of these, 45 articles were selected for full-text review. Full-paper review excluded 33 articles, and 12 studies were finally included in the current meta-analysis. Figure 1 shows the details of the study selection process.

### Study characteristics

The important characteristics of the studies are presented in Table 2. All studies provided information on a per-lesion basis. All studies together included 627 patients with 793 HCC lesions. Most of the studies were retrospective, with three claiming to be prospective. Three studies [11, 14, 16] assessed HCC in patients before liver transplantation, whereas most of the studies included patients suspected of HCC or retrospectively diagnosed with focal liver lesions (FLLs). Eight studies [7, 10, 11, 13, 14, 16, 20, 21] included only patients with cirrhosis. The average lesion size in all studies was approximately 1.6 cm (range, 0.2–15.2 cm). Five studies used only a 64-row CT scanner [7, 11, 12, 14, 16]. Two studies used a 16-row CT scanner [8, 9]. The rest of the studies used a mix of several CT scanners with row numbers ranging from 6 to 64. For MR imaging, six studies used a 3.0-T scanner [10, 11, 13, 15, 16, 21] and six used a 1.5-T scanner [7–9, 12, 14, 20]. Seven studies used a reference standard based solely on histopathology obtained from biopsy, liver resection, or liver transplantation [8, 9, 11, 12, 14–16]. The remaining studies used a composite reference standard that included histopathology and clinical follow-up.

### Evaluation of study quality

**Fig. 2:**
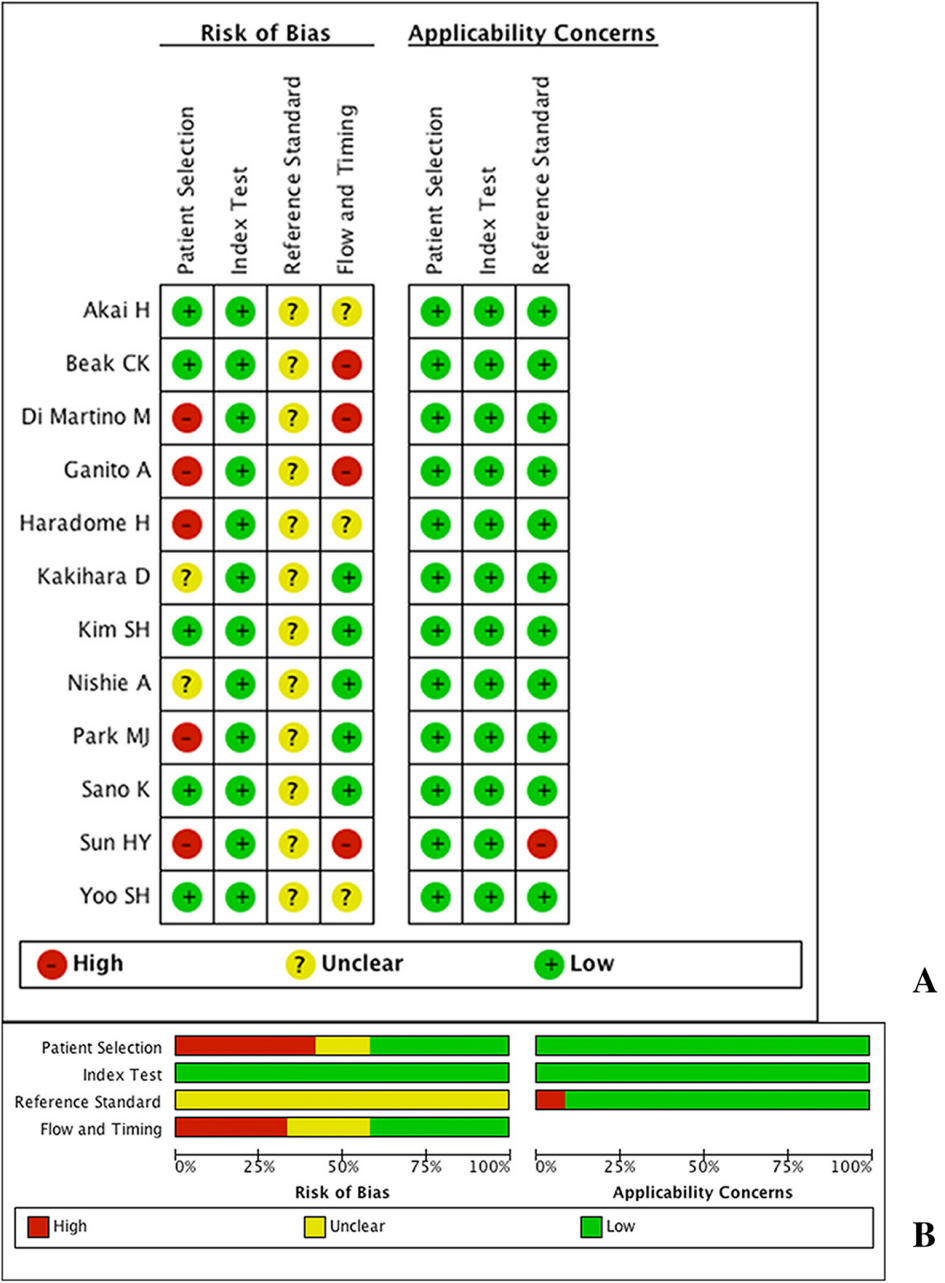
Methodological quality of the 20 included studies assessed with QUADAS-2 tools. **A** Risk of bias; **B** concerns regarding applicability

The distribution of study quality according to the QUADAS-2 tool is shown in Fig. 2. A risk of bias was identified for all domains. Referring to Fig. 2 and Table 2, most studies gave a clear description of participants, index and reference tests, and diagnostic criteria. However, there were different study designs, and often there was a composite standard of reference, which gave rise to the most risk of bias. The risk of bias associated with the Patient Selection domain was attributed to case–control studies, which were defined as studies including both patients with HCC and patients without HCC. Five studies were ranked with high risk of bias due to a case–control study design (*n* = 5) and/or a specified inclusion criteria of patients, and two were ranked unclear due to unclear study design (*n* = 2). All studies were carried out blocking reference information from physicians when they read the images, and all studies contain a clearly pre-specified standard for imaging diagnosis, thus leading to a satisfactory risk of bias in index tests, resulting in a satisfactory. The reference standard domain showed an unclear risk of bias in all studies because of lack of information on whether the pathological analysis was blinded from the results of the index tests. The risk of bias associated with the flow and timing domain was primarily caused by the various reference standards, meaning that not all patients were receiving a single type of reference examination (*n* = 7).

### Diagnostic accuracy

**Fig. 3:**
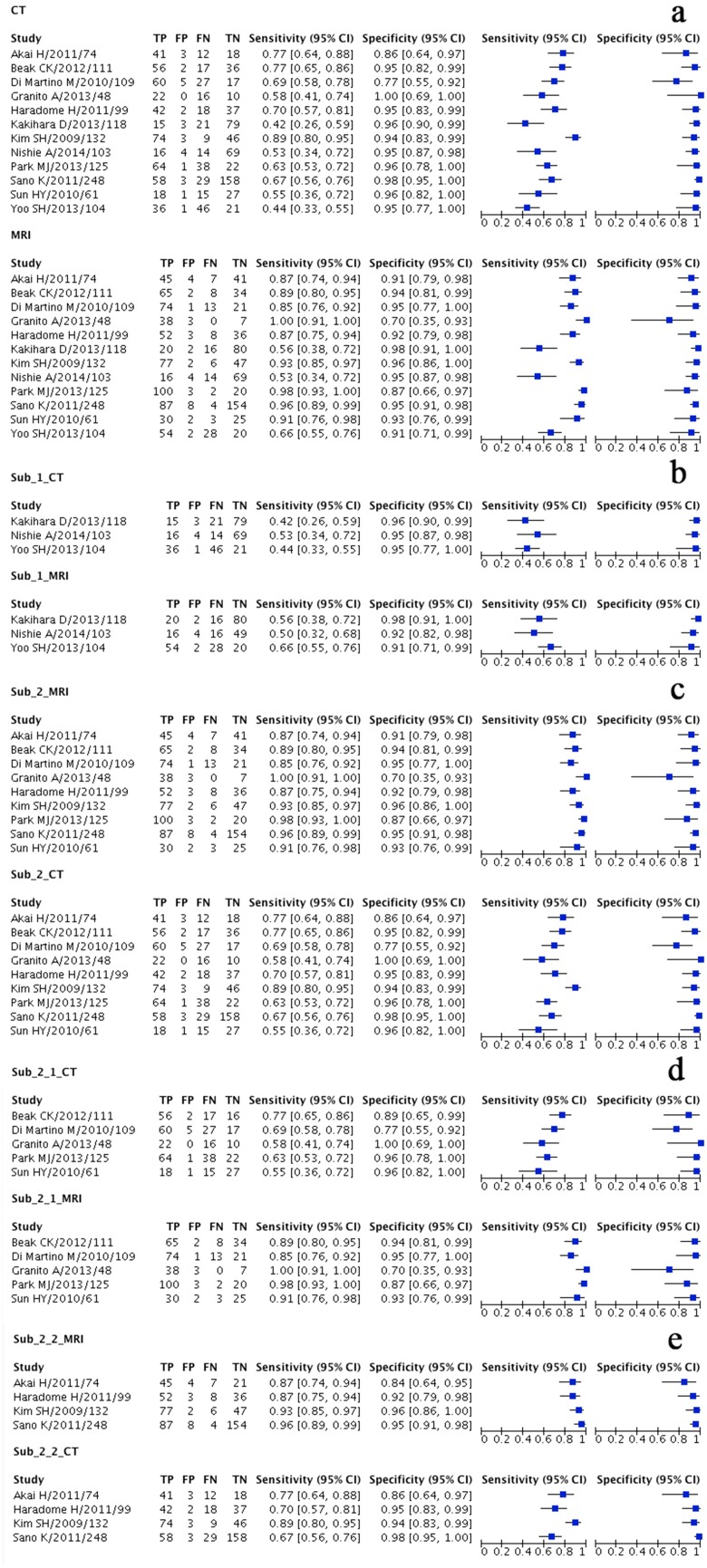
Forest plots showing per-lesion sensitivity and specificity with corresponding 95% Confidence Intervals (CIs) for the diagnosis of HCC by Gd-EOB-DTPA Enhanced MR imaging and multidetector CT in each study. The “Study” on the left was illustrated in the form of “first author/year/sample size”, “sample size” referring to the total number of lesions detected in a single study (please refer to Table 2). **A** Overall analysis; **B** Subgroup of studies using findings in explanted liver as the only reference; **C** Subgroup in which findings in explanted liver were not used as the only reference; **D** Further subgroup of studies in which patients were all diagnosed with cirrhosis; **E** Further subgroup of studies in which patients were partially diagnosed with cirrhosis

**Fig. 4:**
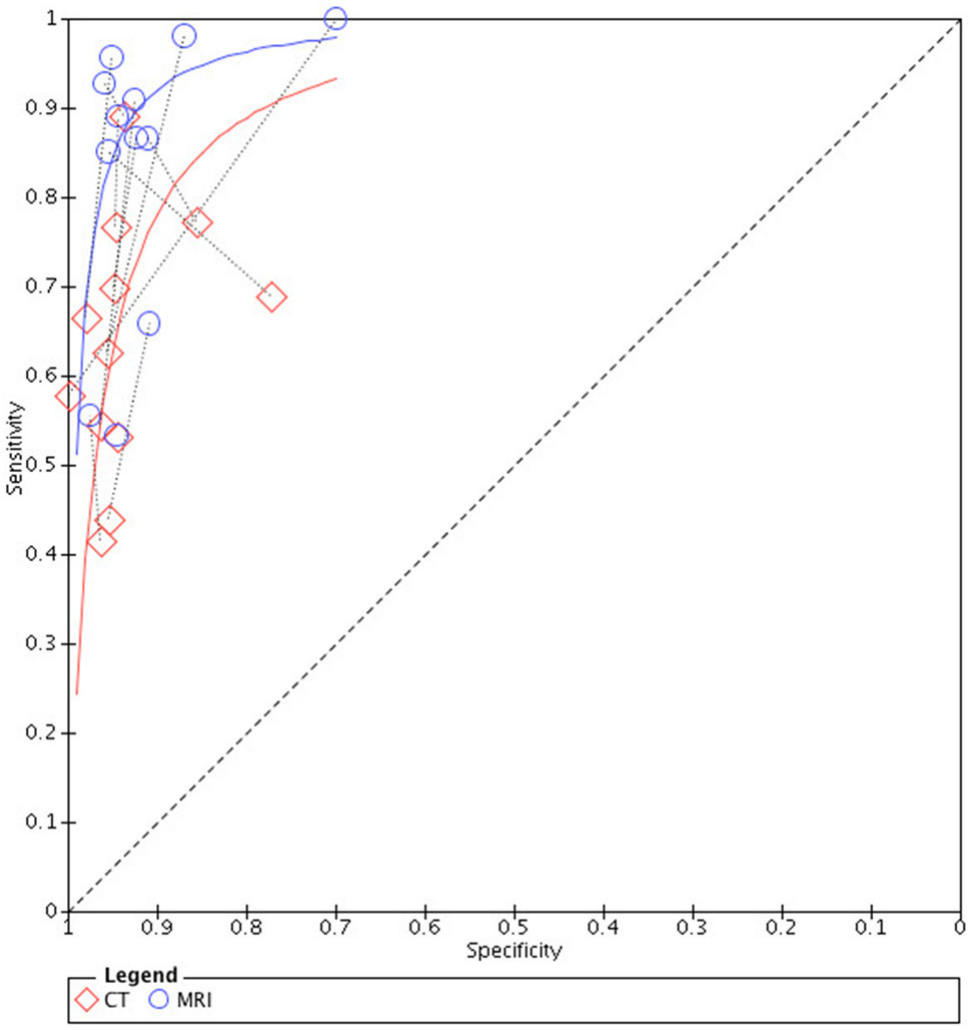
Paired SROC curves of Gd-EOB-DTPA-enhanced MR imaging (MRI) and multidetector CT (CT). A line connects the pair of points representing the tests of MRI and CT from each study

Results are presented in Table 3. The pooled sensitivities of MRI and CT were, respectively, 0.86 (95% CI 0.76–0.93) and 0.70 (95% CI 0.58–0.80). Likelihood ratio tests showed that there is significant difference between the pooled sensitivities of the two tests (*P* < 0.05). The pooled specificities were shown to be similarly high, both 0.94 (95% CI 0.92–0.96) (*P* > 0.05). The summarized PLR of both the tests were larger than 1.00, and the summarized NLRs were smaller than 1.00, indicating informative results of both tests. The forest plots for sensitivities and specificities of Gd-EOB-DTPA-enhanced MR imaging and multidetector CT are shown in Fig. 3, and paired SROC curves for Gd-EOB-DTPA-enhanced MR imaging and multidetector CT are shown in Fig. 4. Paired SROC curves revealed the difference of the diagnostic performance between the two studies: the curve of MRI was formed above that of CT indicating a seemingly larger area under the curve, which is related with the diagnostic ability.

**Table 3:** Results of overall and subgroup analyses, including sensitivity, specificity, AUC, PLR, and NLR

Study	Comparator	Study number	Summarized sensitivity (95% CI^a^)	Chi-square (df^b^)	*P* value	Summarized specificity (95% CI)	Chi-square (df^d^)	Summarized PLR^c^	Summarized NLR^d^	*P* value
Overall analysis										
	MRI^e^	12	0.86 (0.76–0.93)	4.84 (1)	0.03	0.94 (0.92–0.96)	0.04 (1)	14.73 (9.97–21.78)	0.14 (0.08–0.25)	0.80
	CT^f^		0.70 (0.58–0.80)			0.94 (0.92–0.96)		12.87 (8.78–18.87)	0.32 (0.22–0.47)	
Subgroup analysis: findings in liver explantation as the sole reference standard										
Yes	MRI	3^g^	0.61 (0.52–0.69)	4.92 (1)	0.03	0.95 (0.91–0.98)	0.00 (1)	13.42 (6.47–27.84)	0.41 (0.33–0.52)	1.00
	CT		0.45 (0.37–0.53)			0.95 (0.91–0.98)		10.02 (4.97–20.17)	0.57 (0.49–0.66)	
No	MRI	9^h^	0.91 (0.87–0.94)	7.92 (1)	0.00	0.94 (0.90–0.96)	0.09 (1)	14.04 (9.39–21.00)	0.10 (0.07–0.14)	0.76
	CT		0.76 (0.64–0.84)			0.94 (0.89–0.97)		13.04 (9.39–21.00)	0.26 (0.17–0.39)	
Further subgroup analysis of subgroup “Findings in liver explantation NOT as the sole reference standard”: based on whether or not patients were all diagnosed with cirrhosis										
Cirrhosis	MRI	5^i^	0.93 (0.81–0.98)	3.79 (1)	0.05	0.91 (0.81–0.96)	0.10 (1)	10.50 (4.88–22.55)	0.07 (0.02–0.22)	0.75
	CT		0.74 (0.55–0.87)			0.92 (0.85–0.96)		9.90 (5.28–18.55)	0.28 (0.15–0.51)	
Cirrhosis or not	MRI	4^j^	0.91 (0.86–0.94)	6.29 (1)	0.01	0.94 (0.90–0.96)	0.38 (1)	15.14 (9.04–25.34)	0.09 (0.06–0.15)	0.54
	CT		0.77 (0.66–0.86)			0.95 (0.91–0.98)		17.08 (8.62–33.82)	0.24 (0.16–0.35)	

^a^Confidence interval

^b^Degrees of freedom

^c^Positive likelihood ratio

^d^Negative likelihood ratio

^e^Gd-EOB-DTPA-enhanced MR imaging

^f^Contrasted-enhanced multidetector CT

^g^Kakihara et al. [14], Nishie et al. [16], Yoo et al. [11]

^h^Akai et al. [12], Baek et al. [13], Di Martino et al. [7], Granito et al. [20], Haradome et al. [8], Kim et al.[15], Park et al. [21], Sano et al. [9], Sun et al. [10]

^i^Baek et al. [13], Di Martino et al. [7], Granito et al. [20], Park et al. [21], Sun et al. [10]

^j^Akai et al. [12], Haradome et al. [8], Kim et al. [15], Sano et al. [9]

### Subgroup analysis

Meta-regression analysis identified reference standard (whether or not the findings of liver explantation were used as the sole reference standard) as the most important factor responsible for heterogeneity in the two tests; therefore, subgroup analysis was performed based on this factor. The results of subgroup analysis are shown in Table 3. In all the subgroups, specificities of the two tests maintained at a level similar with that of the overall analysis, ranging from 0.91 to 0.95 with no significant difference between them (*P* > 0.05). Sensitivities varied among the subgroups, but the sensitivity of MRI was higher than that of CT in all occasions with or without statistical significance. The PLRs and NLRs remained similar with the results of overall analysis. The forest plots (Fig. 3) also showed subgroup analysis of paired sensitivities and specificities of the two tests.

- Studies in which liver explant findings were used as the sole reference standard were included in one subgroup. There were three studies in this subgroup [11, 14, 16]. The result was in accordance with overall diagnostic performance, though the sensitivities of both the tests were reduced: MRI 0.61 (95% CI 0.52–0.69) and CT 0.45 (95% CI 0.37–0.53). There was significant difference between the sensitivities (*P* < 0.05).
- The second subgroup included studies in which liver explant findings were not the sole reference standard. There were nine studies in this subgroup [7–10, 12, 13, 15, 20, 21]. This time the sensitivity turned out to be significantly higher than that of CT: 0.91 (95% CI 0.87–0.94) v.s. 0.76 (95% CI 0.64–0.84), *P* = 0.00. This subgroup was further divided into two subgroups based on the inclusion of patients to further break down possible heterogeneity. The first subgroup included five studies in which all patients were diagnosed with cirrhosis [7, 10, 13, 20, 21]. The second subgroup included four studies in which only partial patients had cirrhosis [8, 9, 12, 15]. Both of the subgroups showed similar results as the subgroup (b). The pooled sensitivities of MRI were, respectively, 0.91 and 0.93 while those of CT, respectively, 0.74 and 0.77. Significant difference (*P* < 0.05) was found in between the sensitivities of both tests in the second subgroup where the inclusion had not been confined to patients with cirrhosis. In the other subgroup, there was limited difference (*P* = 0.05) between the sensitivities of MRI and CT.

### Lesion size

Size was proved to be an especially important factor for the diagnostic performance of both tests (Table 4). Sensitivities of MRI and CT were reduced as the size of lesion reduced and were particularly low when the lesion was less than 1 cm (MRI 0.46 (95%CI 0.30–0.63), CT 0.20 (95%CI 0.11–0.32)). But the sensitivity of MRI was stably higher than that of CT in all sizes of lesions with or without statistical significance (*P* < 0.05 in lesions ≤ 1 cm and lesions ≤ 2 cm, *P* > 0.05 in lesions > 1 cm and lesions > 2 cm).

**Table 4:** Sensitivity estimates for different lesion sizes

Lesion size	Number of studies	MRI^a^	CT^b^	Chi-square (df^d^)	*P* value
		Sensitivity (95% CI^c^)	Sensitivity (95% CI)		
≤1 cm	4^e^	0.46 (0.30–0.63)	0.20 (0.11–0.32)	4.62 (1)	0.03
>1 cm	6^f^	0.86 (0.73–0.94)	0.74 (0.56–0.87)	1.53 (1)	0.22
≤2 cm	9^g^	0.82 (0.70–0.91)	0.53 (0.36–0.70)	6.41 (1)	0.01
>2 cm	3^h^	0.98 (0.85–1.00)	0.94 (0.71–0.99)	0.56 (1)	0.46

The studies were divided into subgroups based on the size of lesions. Sensitivity estimates were calculated for both imaging techniques and compared in each subgroup

^a^Gd-EOB-DTPA-enhanced MR imaging

^b^Contrasted-enhanced multidetector CT

^c^Confidence interval

^d^Degrees of freedom

^e^Baek et al. [13], Kakihara et al. [14], Kim et al. [15], Yoo et al. [11]

^f^Baek et al. [13], Di Martino et al. [7], Granito et al. [20], Kakihara et al. [14], Kim et al. [15], Yoo et al. [11]

^g^Baek et al. [13], Di Martino et al. [7], Granito et al. [20], Haradome et al. [8], Kakihara et al. [14], Kim et al. [15], Park et al. [21], Sano et al. [9], Sun et al. [10]

^h^Baek et al. [13], Granito et al. [20], Kim et al. [15]

## Discussion

Although MR imaging and CT were compared in previous studies, such comparisons either did not confine to MR imaging with solely Gd-EOB-DTPA as contrast agent, and included merely a limited number of head-to-head comparison studies. Direct comparison is the preferable method to analyze differences between two techniques, as it reduces heterogeneity and provides evidence at a higher level [22]. In addition, per-lesion analysis provides additional information to that provided by per-patient analysis, such as the location of lesions. By searching literature thoroughly focusing on studies of head-to-head comparison and by applying a strictly designed inclusion criteria, we collected twelve studies of quite a considerable number of patients and lesions to perform this meta-analysis, aiming to add to the evidence for the feasible application of either imaging scan to detect HCC.

### Summary of evidence

Our results revealed that in diagnosing HCC MR imaging with Gd-EOB-DTPA showed similarly high specificity of above 0.91 in both overall analysis and subgroup analysis. Specificities of both tests maintained at such a level steadily regardless of factors that could influence diagnosis such as inclusion of patients or disease spectrum. And there was no significant difference between the specificities of the tests in all circumstances. However, subgroup analysis based on lesion size was not available for specificity due to lack of true-negative values. We suppose that size could have a substantial influence on specificity, which should be considered in future studies and clinical application.

Sensitivities of the two tests, different from specificities, varied at multiple levels due to the change of disease spectrum, disease severity, and lesion size. And significant difference was found between the sensitivities of MRI and CT in many instances.

The overall sensitivities of MRI and CT were, respectively, 0.86 and 0.70, suggesting that MRI is a better choice to detect HCC. Subgroup analysis provided further evidence.

When detecting HCC in patients who were diagnosed with HCC by solely liver explant, sensitivities of both MRI and CT were reduced to a great extent (MRI 0.61, CT 0.45), probably due to the fact that patients who underwent liver explant were those with more serious hepatic conditions which could have diminished the diagnostic performance of both tests. Even so, MRI was significantly more sensitive than CT in such cases. Although the fact that only three studies satisfied such a patient inclusion criterion should not be omitted, it still suggested that MRI could be a better choice than CT to detect HCC in patients with more serious liver diseases.

In the other subgroup where not all patients were diagnosed by liver explant, sensitivities of both tests increased, especially that of MRI. Additional subgroup analysis based on whether or not all the included patients had liver cirrhosis showed similar results. In this part of subgroup analyses, the sensitivity of MRI stayed above 0.91, while the sensitivity of CT kept below 0.77.

Moreover, size affected the diagnostic performance of both tests to a large extent. The lower diagnostic accuracy in small HCC lesions can be due to the fact that small HCCs are usually early-stage and often lack the characteristic features of HCC. Early HCCs are often not hypervascular and are supplied mostly by portal venous blood. Therefore, the routine imaging standard of intense arterial uptake followed by a ‘‘washout’’ of contrast in the venous-delayed phases has limited application in early HCCs [23]. Our results indicated that HCC detection efficiency of MRI maintained at a higher level than that of CT for detecting HCC lesions of all sizes, especially those less than 1 cm (sensitivity estimate of MRI versus that of CT: 0.46 versus 0.20) or even larger sizes of less than 2 cm (sensitivity estimate of MRI versus that of CT: 0.82 versus 0.53). Previous studies showed that the sensitivity of MRI for detecting HCC less than 1 cm was 0.46–0.48, whereas that of CT was approximately 0.40 [24, 25]. For lesions smaller than 2 cm, sensitivity of MRI was approximately 0.62, whereas that of CT was approximately 0.40 [25]. Our results showed higher sensitivity of MRI for detecting lesions less than 2 cm and better support the guideline of AASLD in that lesions larger than 1 cm can be directly diagnosed using non-invasive imaging modalities.

### Comparison with previous literature

Although MR imaging and CT were compared in previous studies, a direct comparison of the per-lesion diagnostic performance of Gd-EOB-DTPA-enhanced MR imaging and multidetector CT for HCC had not been performed to date. Therefore, we refer to previous work focusing on the general comparison of MR imaging and CT for HCC. A previous meta-analysis by Lee [24] showed that MR imaging was more sensitive than multidetector CT for the diagnosis of HCC (80% vs. 68%), and MR imaging with Gd-EOB-GDPA showed the highest sensitivity (87%). Our results showed higher sensitivity estimates for Gd-EOB-DTPA-enhanced MR imaging. However, in that study, Gd-EOB-GDPA-enhanced MR imaging was not compared to multidetector CT on a direct per-lesion basis. In a meta-analysis by Chen et al. [26], the performance of MR imaging with liver-specific contrast agents was superior to that of multidetector CT (sensitivity: 0.91 vs. 0.81; specificity: 0.95 vs. 0.93), which is consistent with our results in the subgroup analysis excluding studies that used liver explant findings as the sole reference. However, this research included studies that used either Gd-EOB-DTPA or superparamagnetic iron oxide particles as liver-specific contrast agents. The specific accuracy of Gd-EOB-DTPA-enhanced MR imaging was not analyzed. Studies have suggested that the effects of superparamagnetic iron oxide particle enhanced MR imaging are not comparable to those of Gd-EOB-DTPA-enhanced MR imaging [27]. At the same time, SPIO is not available worldwide since it is not a contrast agent approved by United State Food and Durg Administration, and therefore, its value of application has been limited. [28]

Therefore, the analysis of the two contrast agents together does not accurately reflect the performance of Gd-EOB-DTPA-enhanced MR imaging. Furthermore, this study was published as a postscript, and the important details of the research, such as patient spectrum, imaging techniques, and reference standards, were omitted. Our results were in agreement with those of previous studies showing that MR imaging with liver-specific agents is generally superior to CT for the diagnosis of HCC. Furthermore, our per-lesion and direct comparison results support that MR imaging with Gd-EOB-DTPA is superior to multidetector CT for the detection of HCC. Direct comparison is the preferable method to analyze differences between two techniques, as it reduces heterogeneity and provides evidence at a higher level [22]. In addition, per-lesion analysis provides additional information to that provided by per-patient analysis, such as the location of lesions.

### Lesion size and comparison with previous literature

Subgroup analysis in our study allowed the comparison of the two imaging techniques in lesions of different sizes. In accordance with previous studies, our results suggested that size is a crucial factor in diagnosing HCC. Previous studies showed that for lesions smaller than 1 cm, sensitivity estimate of Gd-EOB-DTPA-enhanced MR imaging was approximately 0.46–0.48, whereas that of CT was approximately 0.40 [24, 25]. For lesions smaller than 2 cm, sensitivity estimate of Gd-EOB-DTPA-enhanced MR imaging was approximately 0.62, whereas that of CT was approximately 0.40 [25]. Our study showed similar sensitivity for lesions smaller than 1 cm (0.46), whereas sensitivity was higher for lesions smaller than 2 cm (0.82) compared with these previous results. The lower diagnostic accuracy in small HCC lesions is due to the fact that small HCCs are usually early-stage and often lack the characteristic features of HCC. Early HCCs are often not hypervascular and are supplied mostly by portal venous blood. Therefore, the routine imaging standard of intense arterial uptake followed by a ‘‘washout’’ of contrast in the venous-delayed phases has limited application in early HCCs [23]. According to the AASLD, lesions smaller than 1 cm should be controlled by ultrasound surveillance, whereas those larger than 1 cm can be directly diagnosed using non-invasive imaging modalities. Therefore, it is crucial to improve the diagnostic performance for HCC lesions smaller than 1 cm. Our results indicated that for smaller HCC lesions, especially for those smaller than 1 cm, the sensitivity of MR imaging with Gd-EOB-DTPA was higher than that of multidetector CT.

### Summary and limitations

Overall, compared with previous studies, our results provided the first meta-analytical direct per-lesion comparison evidence that Gd-EOB-DTPA-enhanced MR imaging was more suitable for diagnosing HCC than multidetector CT. The superiority of Gd-EOB-DTPA-enhanced MR imaging could be attributed to the fact that MR imaging with Gd-EOB-DTPA detects HCCs using a combination of the above multiphasic standard and the enhancement pattern during HBP. Gd-EOB-DTPA is a liver-specific agent that is taken up by hepatocytes. It entry into hepatocytes is mediated by organic anion transporting polypeptides OATP1B1/B3, and its excretion into the bile occurs via the multidrug resistance protein 2 (MRP2). During HBP, most HCCs appear hypointense because OATP1B1/B3 is downregulated and MRP2 is upregulated [6]. Therefore, in hypovascular early HCCs, HBP may assist diagnosis as it does not depend on the vascular pattern.

Our study had several limitations. First, the studies we analyzed included a relatively broad spectrum of patients, including patients suspected of HCC, patients with FLLs, and patients who had previously undergone treatment for HCC. This could lead to bias in patient selection and influence the diagnostic results, as different diagnostic criteria and thresholds are adopted for various populations. Second, a composite reference standard was used in the studies included in the current work, including histopathology and clinical follow-up. However, this might better represent the daily clinical practice. Third, the diagnostic criteria for HCC using MR imaging with Gd-EOB-DTPA varied among the included studies in our work from typical dynamic appearance to a combination of part of the typical dynamic appearance (e.g., only “washout” without intense arterial intake) and HBP hypointensity or HBP hypointensity alone. However, during HBP, 5%–10% of HCCs are iso- or hyperintense relative to the liver because of low or high MRP2 expression [29]. Therefore, the diagnostic performance of HBP hypointensity needs further investigation. Fourth, the imaging technique also varied among the included studies that we analyzed. For example, timing of the delayed phase ranged from 120 to 180 s for Gd-EOB-DTPA-enhanced MR imaging and from 150 to 240 s for CT imaging. This could lead to bias in index tests. Based on the above considerations, we would evaluate the overall study quality in the current research to be medium–high according to a relatively concordant selection of patients, well-designed index tests, acceptable variety of reference standards, and considerate set of index evaluation timing in most studies. Fifth, specificity subgroup analysis failed to carry out due to lack of true-negative values. However, size may have a considerable influence on specificity too. Sixth, papers written in another language generally merely provided an English abstract instead of a full English version and required the relative language ability which unfortunately we do not have. Therefore, the current study only included papers written in English, to avoid risk caused by wrong interpretation of the study and a single opinion due to the language of a co-worker. Future studies should take this into consideration and include as much variety of studies as possible to contribute to the evidence.

## Conclusion

MR imaging with Gd-EOB-DTPA is superior to multidetector CT for the diagnosis of HCC, showing higher sensitivity in more demanding situations such as severe cirrhosis or lesions measuring smaller than 1 cm.

## Electronic supplementary material

Below is the link to the electronic supplementary material.

Supplementary material 1 (DOC 63 kb)

Supplementary material 2 (DOCX 44 kb)

Supplementary material 3 (EPS 1821 kb)

## References

[1] The World Health Organization. GLOBOCAN 2012. Available: http://globocan.iarc.fr/Pages/fact_sheets_canceraspx

[2] Yang JD, Roberts LR (2010). Hepatocellular carcinoma: A global view. Nat Rev Gastroenterol Hepatol.

[3] Bruix J, Sherman M (2011). Management of hepatocellular carcinoma: an update. Hepatology.

[4] European Association For The Study Of The Liver (2012). EASL-EORTC clinical practice guidelines: management of hepatocellular carcinoma. J Hepatol.

[5] Choi JY, Lee JM, Sirlin CB (2014). CT and MR imaging diagnosis and staging of hepatocellular carcinoma: part II. Extracellular agents, hepatobiliary agents, and ancillary imaging features. Radiology.

[6] Van Beers BE, Pastor CM, Hussain HK (2012). Primovist, Eovist: what to expect?. J Hepatol.

[7] Di Martino M, Marin D, Guerrisi A, Baski M, Galati F, Rossi M, Brozzetti S, Masciangelo R, Passariello R, Catalano C (2010). Intraindividual comparison of gadoxetate disodium-enhanced MR imaging and 64-section multidetector CT in the Detection of hepatocellular carcinoma in patients with cirrhosis. Radiology.

[8] Haradome H, Grazioli L, Tinti R, Morone M, Motosugi U, Sano K, Ichikawa T, Kwee TC, Colagrande S (2011). Additional value of gadoxetic acid-DTPA-enhanced hepatobiliary phase MR imaging in the diagnosis of early-stage hepatocellular carcinoma: comparison with dynamic triple-phase multidetector CT imaging. J Magn Reson Imaging.

[9] Sano K, Ichikawa T, Motosugi U, Sou H, Muhi AM, Matsuda M, Nakano M, Sakamoto M, Nakazawa T, Asakawa M, Fujii H, Kitamura T, Enomoto N, Araki T (2011). Imaging study of early hepatocellular carcinoma: usefulness of gadoxetic acid-enhanced MR imaging. Radiology.

[10] Sun HY, Lee JM, Shin CI, Lee DH, Moon SK, Kim KW, Han JK, Choi BI (2010). Gadoxetic acid-enhanced magnetic resonance imaging for differentiating small hepatocellular carcinomas (< or =2 cm in diameter) from arterial enhancing pseudolesions: special emphasis on hepatobiliary phase imaging. Invest Radiol.

[11] Yoo SH, Choi JY, Jang JW, Bae SH, Yoon SK, Kim DG, Yoo YK, Rha SE, Lee YJ, Jung ES (2013). Gd-EOB-DTPA-enhanced MRI is better than MDCT in decision making of curative treatment for hepatocellular carcinoma. Ann Surg Oncol.

[12] Akai H, Kiryu S, Matsuda I, Satou J, Takao H, Tajima T, Watanabe Y, Imamura H, Kokudo N, Akahane M, Ohtomo K (2011). Detection of hepatocellular carcinoma by Gd-EOB-DTPA-enhanced liver MRI: comparison with triple phase 64 detector row helical CT. Eur J Radiol.

[13] Baek CK, Choi JY, Kim KA, Park MS, Lim JS, Chung YE, Kim MJ, Kim KW (2012). Hepatocellular carcinoma in patients with chronic liver disease: a comparison of gadoxetic acid-enhanced MRI and multiphasic MDCT. Clin Radiol.

[14] Kakihara D, Nishie A, Harada N, Shirabe K, Tajima T, Asayama Y, Ishigami K, Nakayama T, Takayama Y, Okamoto D, Fujita N, Kishimoto J, Honda H (2013). Performance of gadoxetic acid-enhanced MRI for detecting hepatocellular carcinoma in recipients of living-related-liver-transplantation: Comparison with dynamic multidetector row computed tomography and angiography-assisted computed tomography. J Magn Reson Imaging.

[15] Kim SH, Lee J, Kim MJ, Jeon YH, Park Y, Choi D, Lee WJ, Lim HK (2009). Gadoxetic acid-enhanced MRI versus triple-phase MDCT for the preoperative detection of hepatocellular carcinoma. AJR Am J Roentgenol.

[16] Nishie A, Kakihara D, Asayama Y, Ushijima Y, Takayama Y, Fujita N, Shimamoto D, Shirabe K, Hida T, Honda H (2014) Detectability of hepatocellular carcinoma on gadoxetic acid-enhanced MRI at 3T in patients with severe liver dysfunction: clinical impact of dual-source parallel radiofrequency excitation. Clin Radiol 10.1016/j.crad.2014.11.006 25522901

[17] Moher D, Liberati A, Tetzlaff J, Altman DG (2010). Preferred reporting items for systematic reviews and meta-analyses: the PRISMA statement. Int J Surg (London, England).

[18] Whiting PF, Rutjes AW, Westwood ME, Mallett S, Deeks JJ, Reitsma JB, Leeflang MM, Sterne JA, Bossuyt PM (2011). QUADAS-2: a revised tool for the quality assessment of diagnostic accuracy studies. Ann Intern Med.

[19] Van Houwelingen HC, Zwinderman KH, Stijnen T (1993). A bivariate approach to meta-analysis. Stat Med.

[20] Granito A, Galassi M, Piscaglia F, Romanini L, Lucidi V, Renzulli M, Borghi A, Grazioli L, Golfieri R, Bolondi L (2013). Impact of gadoxetic acid (Gd-EOB-DTPA)-enhanced magnetic resonance on the non-invasive diagnosis of small hepatocellular carcinoma: a prospective study. Aliment Pharmacol Ther.

[21] Park MJ, Kim YK, Lee MH, Lee JHWJ (2013). Validation of diagnostic criteria using gadoxetic acid-enhanced and diffusion-weighted MR imaging for small hepatocellular carcinoma ((less-than or equal to) 2.0 cm) in patients with hepatitis-induced liver cirrhosis. Acta Radiol.

[22] Leeflang MM, Deeks JJ, Gatsonis C, Bossuyt PM (2008). Systematic reviews of diagnostic test accuracy. Ann Intern Med.

[23] Kojiro M (2009). Pathologic diagnosis of early hepatocellular carcinoma: a report of the international consensus group for hepatocellular neoplasia. Hepatology.

[24] Lee YJ, Lee JM, Lee JS, Lee HY, Park BH, Kim YH, Han JK, Choi BI (2015). Hepatocellular carcinoma: diagnostic performance of multidetector ct and mr imaging-a systematic review and meta-analysis. Radiology.

[25] Yu MH, Kim JH, Yoon JH, Kim HC, Chung JW, Han JK, Choi BI (2014). Small (</=1-cm) hepatocellular carcinoma: diagnostic performance and imaging features at gadoxetic acid-enhanced MR imaging. Radiology.

[26] Chen L, Zhang L, Bao J, Zhang J, Li C, Xia Y, Huang X, Wang J (2013). Comparison of MRI with liver-specific contrast agents and multidetector row CT for the detection of hepatocellular carcinoma: a meta-analysis of 15 direct comparative studies. Gut.

[27] Kim YK, Kim CS, Han YM, Park G, Hwang SB, Yu HC (2010). Comparison of gadoxetic acid-enhanced MRI and superparamagnetic iron oxide-enhanced MRI for the detection of hepatocellular carcinoma. Clin Radiol.

[28] Hennedige T, Kundapur Venkatesh S (2012). Imaging of hepatocellular carcinoma: diagnosis, staging and treatment monitoring. Cancer Imaging.

[29] Tsuboyama T, Onishi H, Kim T, Akita H, Hori M, Tatsumi M, Nakamoto A, Nagano H, Matsuura N, Wakasa K, Tomoda K (2010). Hepatocellular carcinoma: hepatocyte-selective enhancement at gadoxetic acid-enhanced MR imaging–correlation with expression of sinusoidal and canalicular transporters and bile accumulation. Radiology.

