# Supplementary material for: Diagnostic performance of contrast-enhanced multidetector computed tomography and gadoxetic acid disodium-enhanced magnetic resonance imaging in detecting hepatocellular carcinoma: direct comparison and a meta-analysis
Source: Abdom Radiol (NY). 2016 Jun 18;41(10):1960–72. doi: 10.1007/s00261-016-0807-7 (PMC5018023; doi:10.1007/s00261-016-0807-7)
Supplement: Supplementary file 2 — Supplementary material 2 (DOCX 44 kb) [file 261_2016_807_MOESM2_ESM.docx]

**Supplementary Figure 1.** The pairs of sensitivity and specificity values for Gd-EOB-DTPA Enhanced MR Imaging and Multidetector CT to detect HCC in SROC curves. A, Overall analysis; B, Subgroup of studies using findings in explanted liver as the only reference; C, Subgroup in which findings in explanted liver were not used as the only reference; D, Further subgroup of studies in which patients were all diagnosed with cirrhosis; E, Further subgroup of studies in which patients were partially diagnosed with cirrhosis.
